# Redox Homeostasis and Antioxidant Response After Bariatric Surgery in Severe Obesity: Insights from a Controlled Clinical Cohort

**DOI:** 10.3390/medicina61101884

**Published:** 2025-10-21

**Authors:** Razvan Marius Ion, Erzsébet Májai, Mircea Dumitru Croitoru, Oana Axina Rusti, Gabriela Beresescu, Ibolya Fülöp, Radu Mircea Neagoe

**Affiliations:** 1 Doctoral School of Medicine and Pharmacy, Second Department of Surgery, George Emil Palade University of Medicine, Pharmacy, Science and Technology of Târgu Mures, Mures County Emergency Hospital, 540142 Târgu Mures, Romania; razvan.ion@umfst.ro; 2 Center for Advanced Medical and Pharmaceutical Research, George Emil Palade University of Medicine, Pharmacy, Science and Technology of Târgu Mures, 540142 Târgu Mures, Romania; 3 Department of Toxicology and Biopharmacy, Faculty of Pharmacy, George Emil Palade University of Medicine, Pharmacy, Science and Technology of Târgu Mures, 540142 Târgu Mures, Romania; 4 Second Department of Surgery, George Emil Palade University of Medicine, Pharmacy, Science and Technology of Târgu Mures, 540139 Târgu Mures, Romania; 5 Department of Morphology of Teeth and Dental Arches, Faculty of Dentistry, George Emil Palade University of Medicine, Pharmacy, Science and Technology of Târgu Mures, 540142 Târgu Mures, Romania

**Keywords:** oxidative stress biomarkers, obesity, bariatric surgery, malondialdehyde, glutathione, nitric oxide, nitrite, nitrate

## Abstract

*Background and Objectives:* Obesity represents a major public health concern worldwide, particularly in economically challenged regions, and is often associated with metabolic comorbidities such as type 2 diabetes mellitus, hypertension, and metabolic-associated fatty liver disease. Oxidative stress plays a central role in obesity pathophysiology through the accumulation of reactive oxygen and nitrogen species. This study aimed to investigate changes in specific oxidative stress biomarkers in patients with obesity before and one year after bariatric surgery, with a lean control group as reference. *Methods*: This observational cohort study included 50 patients with morbid obesity undergoing bariatric surgery (laparoscopic sleeve gastrectomy or one-anastomosis gastric bypass) and 50 patients without obesity undergoing other surgical procedures. Plasma levels of malondialdehyde (MDA), reduced and oxidized glutathione (GSH and GSSG), nitrite (NO_2_^−^), and nitrate (NO_3_^−^) were measured preoperatively and one year postoperatively in the bariatric group, and once in the control group. Quantification was performed using HPLC-based techniques. *Results*: Postoperative analysis revealed a significant reduction in oxidative stress markers. MDA levels decreased from 21.58 to 16.62 ng/mL after surgery, while GSH levels increased significantly, although they remained lower than in the control group. GSH/GSSG ratio improved slightly, indicating enhanced antioxidant capacity. Nitrite and nitrate levels showed a marked reduction postoperatively, which may reflect both diminished NO production and complex metabolic adaptations following weight loss. Correlation analysis showed that reductions in BMI were significantly associated with increases in GSH levels and decreases in MDA. *Conclusions*: Bariatric surgery led to significant improvements in key oxidative stress biomarkers in patients with obesity, supporting the hypothesis that weight loss mitigates oxidative damage. However, the reduction in nitrite suggests potential trade-offs in nitric oxide metabolism that warrant further investigation. Long-term studies are needed to determine the clinical significance and sustainability of these biochemical improvements.

## 1. Introduction

Worldwide, the prevalence and incidence of obesity is alarming, especially in countries facing financial difficulties [1]. Most of the time, an individual struggling with obesity suffers from a range of other comorbidities, such as type 2 diabetes (T2DM), dyslipidemia and hypertension (HTN), obstructive sleep apnea (OSA), or metabolic-associated fatty liver disease (MAFLD) [2]. The health complications brought on by obesity leads to an increased risk of premature death, cardiovascular disease, mental health concerns, reproductive and fertility issues, kidney disease, and musculoskeletal problems [3].

In 1985 the concept of oxidative stress was introduced as an imbalance between production in the human body of the free radicals and its ability to trap them into stable compounds. This results either from an increased production of reactive species such as ROS (reactive oxygen species), RNS (reactive nitrogen species), or other free radicals centered on other atoms such as S, C, etc. [4]. This imbalance between the production and elimination of free radicals and other reactive species—relatively stable, non-radicalic compounds that can be converted into free radicals under certain conditions—plays a critical role in the pathophysiology of oxidative stress [5].

Oxidative stress arises from an imbalance between the production of reactive oxygen species (ROS) and the body’s antioxidant defenses. Glutathione (GSH), a tripeptide containing cysteine, glutamic acid, and glycine, is one of the most important antioxidants. It neutralizes ROS by donating electrons via its thiol group, becoming oxidized to GSSG in the process. Superoxide dismutases (SOD) convert superoxide anions into hydrogen peroxide, which may further generate highly reactive hydroxyl radicals (•OH). These radicals can trigger lipid peroxidation, cellular damage, and inflammation [6]. Malondialdehyde (MDA) is widely used as a marker of oxidative stress caused by •OH radicals [7]. Nitric oxide (NO), another key signaling molecule, regulates vascular tone, blood flow, oxygen delivery, and muscle function. Its bioavailability is often assessed by measuring nitrite (NO_2_^−^), while dietary nitrate (NO_3_^−^) from vegetables can also contribute to NO production through the nitrate–nitrite–NO pathway [8].

In previous work, we have focused on elucidating the relationship between chronic systemic inflammation and weight-reduction outcomes following laparoscopic sleeve gastrectomy (LSG), emphasizing the utility of plasma cytokine profiling as a biomarker of inflammatory status [9,10]. Another study investigated the link between ROS, obesity, and metabolic syndrome to evaluate whether bariatric surgery-induced weight loss mitigates obesity-related inflammation and metabolic comorbidities [11].

The purpose of this study was to evaluate if obesity is associated with oxidative stress biomarkers such as MDA, GSH, GSH/GSSG, total glutathione levels, NO_2_^−^, and NO_3_^−^ in patients with obesity before and after being subjected to bariatric surgery and to follow the changes brought by such surgery. A group of patients without obesity served as reference for normal values.

## 2. Materials and Methods

### 2.1. Study Design

We established a prospective observational cohort at two centers in Târgu Mureș, Romania, enrolling 50 adults with morbid obesity undergoing bariatric surgery and 50 non-obese surgical patients as controls (enrollment between 1 February 2021–1 December 2023). Bariatric participants were sampled preoperatively (B1) and 12 months postoperatively (B2); thus, B1 and B2 represent the same individuals. Inclusion and exclusion criteria, comorbidities, and perioperative care followed established guidance. All participants provided written informed consent.

Sub-objectives of this study were to assess the evolution of inflammatory and oxidative stress biomarkers among severely obese subjects undergoing LSG. Thereby, we submit a comprehensive survey of some of the most important OS-status biomarkers in the morbidity of obese individuals, both at baseline and 1 year following bariatric surgery, in comparison with baseline values from patients undergoing non-obesity-related surgery procedures.

**Inclusion Criteria**

- Adults with severe obesity eligible for LSG
- Complete medical records available
- Consent to participate in the study

**Exclusion Criteria**

- Malignancies
- Acute inflammatory diseases
- Severe blood or autoimmune disorders
- Major mental health conditions
- Overweight or first-degree obesity patients
- Chronic inflammatory conditions (e.g., Crohn’s disease, chronic pancreatitis)
- Ongoing use of anti-inflammatory, antibiotic, antiviral, or other long-term drug therapies
- Active alcohol or illicit drug abuse
- Incomplete medical records
- Laboratory-confirmed COVID-19 infection at the time of surgery (all patients were tested preoperatively)

Each patient received full information about the purpose of the study and the procedures to be followed and gave their consent. Approval for the study was obtained from the committees on ethics of the two hospitals involved, Mures County Emergency Hospital (protocol code 3570, on 19 February 2021) and Topmed Private Hospital Târgu Mureș (registration number 114, on 16 March 2021), to ensure that the principles set out in the Helsinki Declaration are strictly followed.

### 2.2. Clinical Assessments

Patients were assessed preoperatively by a multidisciplinary team, covering medical history gathering, physical examination and laboratory tests. Anthropometric measurements were performed preoperatively which included body height (BH), precision on measurement +/− 0.5 cm, body weight (BW), precision on measurement +/− 0.1 kg, and waist circumference (WC) measured on the midline between the anterior superior iliac crest and the lowest rib. Their body mass index (BMI) levels were calculated based on weight (expressed in kilograms) divided by the square of the body height (measured in meters). Blood pressure and heart rate values were obtained with the use of a semi-automatic oscillometric device, following standard recommendations. In addition, we recorded relevant clinical data (comorbidities like hypertension, diabetes, dyslipidaemia) as well as laboratory data from routine patient assessment (for example, lipid profiles) [12].

### 2.3. Oxidative Stress Markers and Antioxidants Assessment

The following markers of oxidative stress were measured before surgical intervention and one year after: malondialdehyde, reduced and oxidized glutathione, nitrite, and nitrate anions.

#### 2.3.1. Reduced and Oxidized Glutathione Measurement

Quantification of GSH and GSSG was carried out according to the method of Jîtcă G. et al., based on the reaction with Ellman’s reagent (DTNB). In this assay, reduced glutathione reacts with DTNB to produce a chromophore detected at 330 nm. Total glutathione was determined after chemical reduction of oxidized forms, while GSSG was calculated from the difference between total glutathione and GSH in Table 1. Care was taken to avoid hemolysis, as erythrocytes contain markedly higher glutathione concentrations than plasma, which could bias results. Analyses were performed on a quaternary HPLC system equipped with a diode array detector (Merck, Darmstadt, Germany). Calibration curves were established using certified standards of GSH and GSSG [12].

#### 2.3.2. Malondialdehyde Measurement

MDA levels were assessed using the thiobarbituric acid-reactive substances (TBARS) assay coupled with HPLC, as previously described by Fogarasi E et al. This approach provides greater specificity than simple spectrophotometric detection. 1,1,3,3-Tetramethoxypropane (TMP) was used as a stable precursor for MDA calibration. Chromatographic detection was performed at 532 nm, with isocratic elution of acetonitrile and phosphate buffer (pH 6.0) [13].

#### 2.3.3. Nitrite and Nitrate Measurement

Nitrite and nitrate concentrations were determined simultaneously by HPLC with UV/VIS detection following the method of Croitoru et al. Nitrite was quantified through the Griess reaction, producing an azo dye detectable at 520 nm, while nitrate was measured by ion-pair chromatography with tetrabutylammonium hydroxide as the pairing agent in Table 2. Calibration was performed with sodium nitrite and sodium nitrate standards [14].

### 2.4. Surgical Intervention Protocol

Patients with obesity underwent two kinds of bariatric interventions: most of them underwent laparoscopic sleeve gastrectomy (LSG), with the steps of the procedure previously reported by us [9], without significant technical differences. A minority of patients among this group (i.e., the bariatric group) underwent laparoscopic gastric bypass with a single anastomosis (OAGB), where a gastrojejunostomy was performed 180 cm below the duodenojejunal angle in a side-to-side antecolic manner with either a linear or manual stapler. The procedures carried out in the control group were the following: partial or total thyroidectomy, laparoscopic cholecystectomy, transabdominal preperitoneal or total extraperitoneal inguinal hernia repair.

### 2.5. Follow-Up

During the first year, we prospectively followed all our patients for 3 months post-surgery. Records were gathered during postoperative follow-up appointments; we recorded the weight-loss process, influence of the bariatric procedure over the main comorbidities of obesity and other clinical as well as technical matters linked to the procedure. All bariatric participants were sampled at 12 months for biomarker reassessment.

### 2.6. Analytical Validation and Reproducibility

All biomarker determinations were performed using HPLC-based techniques with UV or VIS detection, as detailed above. Reduced and oxidized glutathione (GSH and GSSG) were quantified using Ellman’s reagent with calibration against certified standards, while malondialdehyde (MDA) was measured via the thiobarbituric acid reaction using 1,1,3,3-tetramethoxypropane as a stable standard. Nitrite and nitrate were determined by the Griess reaction combined with ion-pair chromatography. The HPLC system consisted of a quaternary pump, column thermostat, diode array detector, and autosampler (Merck, Darmstadt, Germany). Calibration curves for all analytes showed excellent linearity (R^2^ > 0.99). Samples were measured in duplicate, and intra-assay coefficients of variation were <10% for all assays. To ensure data reliability, hemolyzed samples were excluded due to the high intracellular glutathione content in erythrocytes. Analytical reproducibility and accuracy were confirmed by repeat analyses of randomly selected samples and through comparison with published methods [13,14,15].

### 2.7. Statistical Analysis

Sample size justification: The study included 50 patients with obesity and 50 normoponderal controls. Although no formal a priori power calculation was performed, this sample size is comparable to or larger than those used in previous clinical studies investigating oxidative stress biomarkers in obesity and bariatric cohorts [15]. Therefore, it was considered sufficient to detect clinically meaningful differences within the analyzed parameters.

All statistical analyses were performed using GraphPad Prism 9 (GraphPad Software, Inc.), Microsoft Excel, and OriginPro 2018. Continuous variables were expressed as mean ± standard deviation (SD). Data distribution was assessed using the Shapiro–Wilk test to determine normality. Depending on the data distribution, either parametric tests (e.g., paired or unpaired two-tailed Student’s *t*-test) or non-parametric tests (e.g., Wilcoxon signed-rank test, Mann–Whitney U test) were applied for within-group and between-group comparisons, respectively. One-tailed *t*-tests were used in cases where a directional hypothesis was justified based on prior literature or expected biological outcomes.

Correlation analyses were conducted using Pearson’s correlation coefficient (for parametric data) or Spearman’s rank correlation coefficient (for non-parametric data), with respective *p*-values reported. Repeated measures ANOVA was used where applicable to assess changes over time in the bariatric group.

Statistical significance was considered at *p* < 0.05, and all tests were two-tailed unless otherwise specified. Confidence intervals (95%) were calculated where appropriate. Statistical outputs were visualized using bar plots, scatter plots with regression lines, and box-and-whisker plots.

## 3. Results

In our test we measured the concentration of the selected biomarkers [malondialdehyde (MDA), reduced and oxidized glutathione (GSH and GSSG), nitrite (NO_2_^−^), and nitrate (NO_3_^−^)] to evaluate the effects of bariatric surgery on the oxidative parameters of patients with obesity.

Both the obese and normoponderal groups included 50 participants each. While no formal a priori power analysis was performed, the sample size is in line with or exceeds that reported in comparable studies on oxidative stress in obesity [16] and was considered adequate for detecting relevant group differences.

ANOVA test was used to compare the means of a continuous outcome across more than two groups and Chi-square test was used to compare the distribution of a categorical variable across multiple groups. Data are presented as mean ± standard deviation (SD). All abbreviations used in this Table 3 are defined at the end of the article in the Abbreviations section.

Laboratory parameters such as glucose, liver enzymes (GOT, GPT, GGT), hemoglobin, hematocrit, leukocytes and their subpopulations (neutrophils, lymphocytes, eosinophils), as well as platelet count, were included to provide a comprehensive metabolic and clinical profile of the patients. These markers reflect systemic inflammation, hepatic function, and metabolic status, all of which are commonly altered in obesity and can influence oxidative stress levels. Their inclusion allowed for a more accurate interpretation of redox biomarkers by accounting for potential confounding factors that could impact oxidative stress independently of the bariatric intervention.

As expected, the reduction in the intensity of the metabolic processes brought about by the low calorie intake that follows a bariatric surgery took place, and our measurements proved this through tests for the reduction in free radical formation.

We measured malondialdehyde (MDA) as the main free radical formed in the human body in both obese and normoponderal subjects. It is formed during HO reaction with polyunsaturated fatty acids—cell membrane being the most sensitive cell component.

The expected outcome of our study was the modification of all of our measured parameters: MDA, GSH, GSSG and their ratio (GSH/GSSG), nitrite, and nitrate and their ratio.

### 3.1. Glutathione Values

Free radical formation was indeed commonly reduced following bariatric surgical treatment.

**Figure 1:**
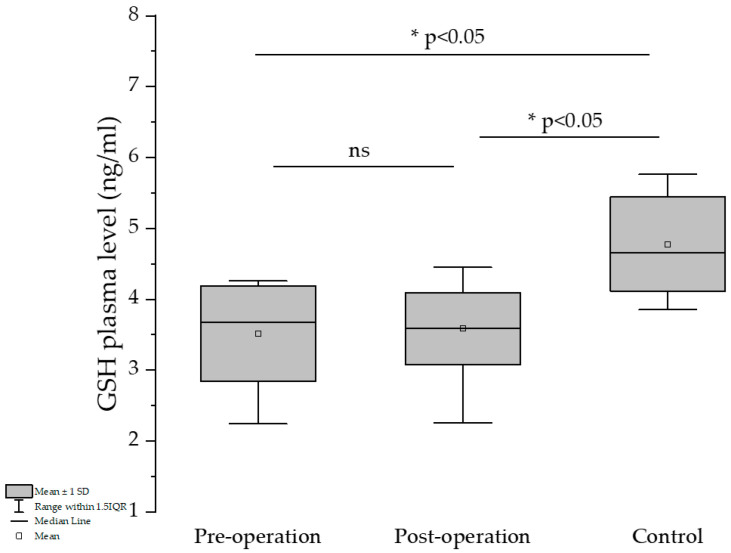
Evaluation of GSH before and one year after bariatric surgery, and control group. * indicates a statistically significant difference (*p* < 0.05); “ns” indicates no significant difference (*p* ≥ 0.05).

Comparing the pre- and post-operation groups, a slight GSH-level elevation could be observed for the latter, but the differences are not significant (Figure 1).

The percentage of GSH was slightly increased in the post-surgery group compared to the pre-treatment group and almost attained the control group’s GSH-level percentage (42.7 and 43.7 vs. 47.4%). The GSH/GSSG ratio is below 1 in all three groups, which can be explained by the fact that all of them suffered under different pathological conditions.

As expected, increase in BMI reduction (ΔBMI) was inversely associated with the decrease in the Δ GSH values, which was revealed by a one-tailed *t*-test—this type of test was used because it was expected that an increase in body weight reduction would increase production of GSH, and only that hypothesis was tested. Furthermore, the reduction in BMI is inversely correlated with GSH values (r = −0.2526, p* = 0.0384, one-tailed *t*-test), showing the beneficial effects of bariatric surgery on the protective-antioxidant effect on the increase in GSH values. It is worth mentioning that preoperatory values of GSH in patients with obesity were significantly lower than those measured in the control group (3.513 vs. 4.778, unpaired two-tailed *t*-test, *p* < 0.0001 ****) but were almost the same as values recorded after surgery, which were still statistically lower than values recorded for the control group (3.586 vs. 4.778, unpaired two-tailed test, *p* < 0.0001 ***).

Additionally, our measurements showed that decreases in BDW induce less increases in GSH values (r = −0.256, one-tailed *t*-test, p* = 0.0384).

### 3.2. Malondialdehyde Values

**Figure 2:**
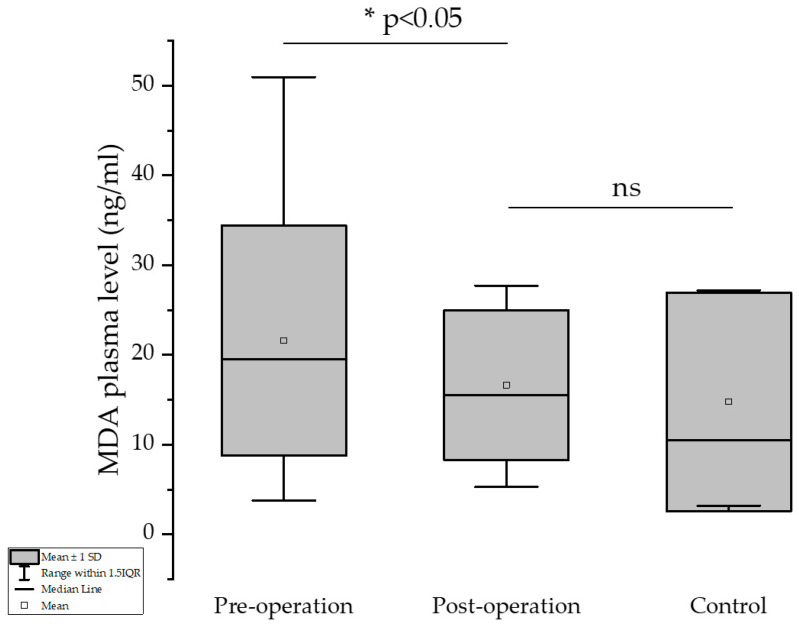
Evaluation of MDA before and one year after bariatric surgery, and control group. * indicates a statistically significant difference (*p* < 0.05); “ns” indicates no significant difference (*p* ≥ 0.05).

The results showed that excessive formation of MDA due to increased metabolism and excessive formation of its precursors (mainly H_2_O_2_) was significantly reduced after the bariatric surgery procedure. The higher the loss of body weight (expressed as difference in BMI pre- and one-year post-surgical intervention), the higher the decrease in the MDA values (Pearson’s test, −0.25588, p* = 0.0348). Mean of the MDA values pre- and post-operation were 21.58 and 16.62 ng/mL, respectively, showing a significant decrease in this significant marker of the oxidative stress (*p* = 0.013, one-tailed paired *t*-test). There was no significant difference between the post-operation and control groups (*p* > 0.05, one-sample *t*-test) in terms of MDA plasma level (Figure 2).

### 3.3. Nitrite and Nitrate Values

**Figure 3:**
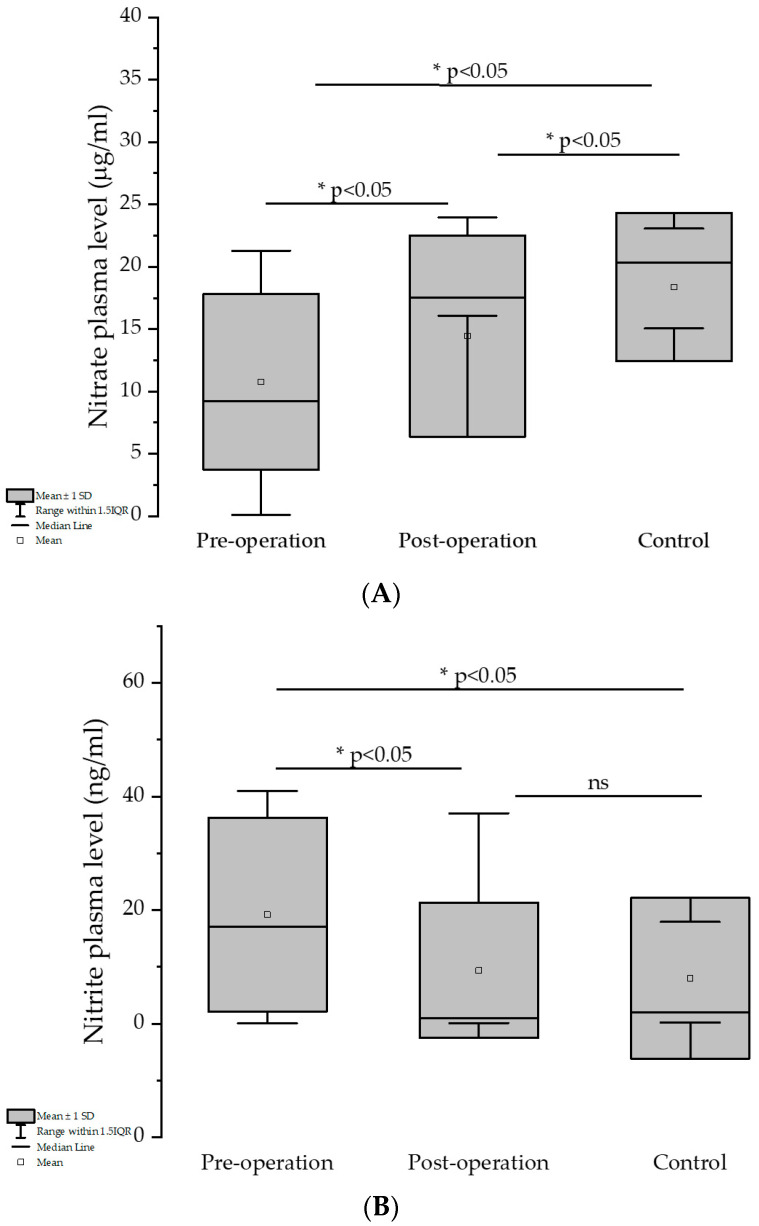
(**A**,**B**) Evaluation of Nitrate and Nitrite before and one year after bariatric surgery, and control group. * indicates a statistically significant difference (*p* < 0.05); “ns” indicates no significant difference (*p* ≥ 0.05).

Regarding the nitrate values, the mean values increased in the order of pre-, post-operation and control group, showing significant differences in all cases in Figure 3A (*p* < 0.05, using paired *t*-test and two-sample *t*-test, respectively).

In the case of nitrite, the mean value of the post-operation group was close to that obtained in the control group (9.40 and 8.01 ng/mL, respectively), the difference not being statistically significant (two-sample *t*-test). Regarding the pre-operation group’s mean nitrite value, a significant decrease can be observed compared to that of the one-year post-operation group in Figure 3B (19.22 vs. 9.40 ng/mL, *p* < 0.05, paired *t*-test).

**Figure 4:**
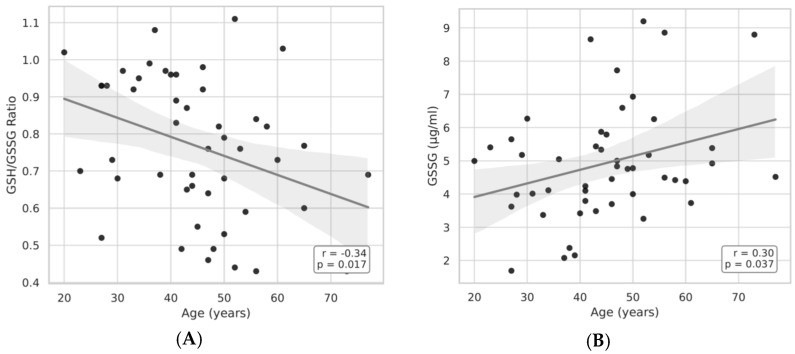
(**A**,**B**) Pearson’s correlation between age and GSSG-GSH/GSSG in the B1 group.

As shown in Figure 4A,B, age was significantly correlated with both the GSH/GSSG ratio and plasma GSSG levels. Specifically, the GSH/GSSG ratio decreased with increasing age (r = –0.34, *p* = 0.017), while GSSG concentrations rose slightly with age (r = 0.30, *p* = 0.037). These findings indicate an age-related decline in redox balance.

**Figure 5:**
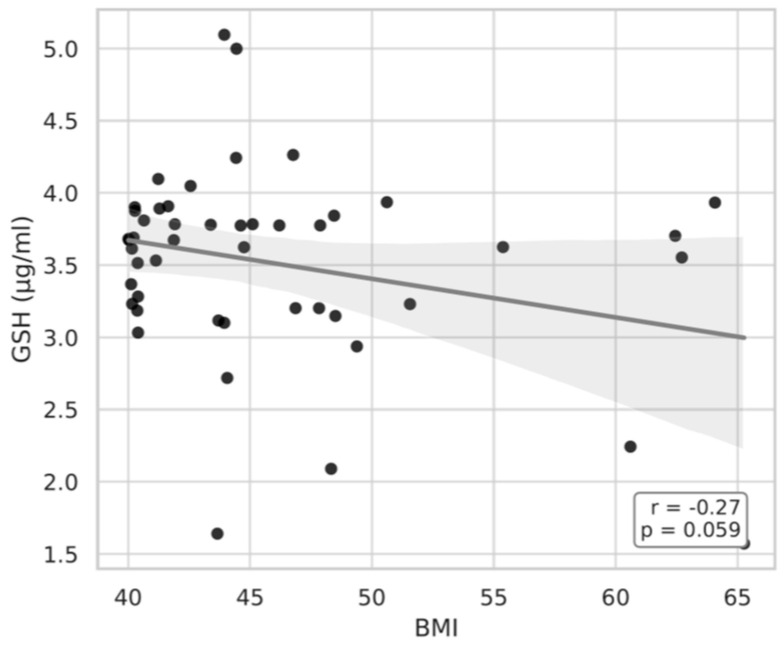
Pearson correlation between BMI and GSH in the B1 group.

**Figure 6:**
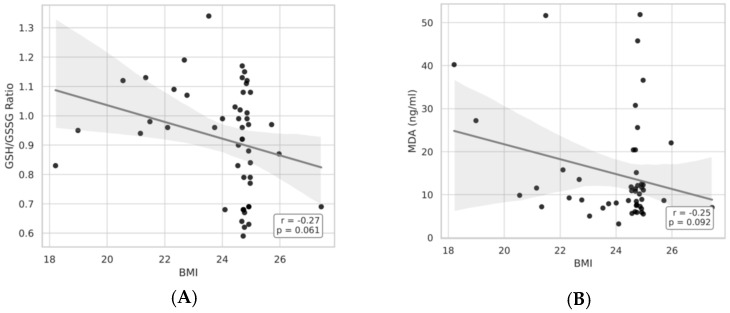
(**A**,**B**) Pearson’s correlation between BMI and MDA—GSH/GSSG in the B1 group.

As illustrated in Figure 5 and Figure 6A,B, BMI showed significant correlations with both the GSH/GSSG ratio, plasma GSH and MDA concentrations. In particular, the GSH/GSSG ratio declined with advancing BMI (r = −0.27, *p* = 0.061), whereas plasma GSH levels exhibited a modest decrease (r = −0.27, *p* = 0.059). Together, these results suggest an bmi-associated reduction in redox homeostasis.

## 4. Discussion

Obesity and oxidative stress are correlated based on the data in the scientific literature [16]. In our study, several markers of oxidative stress were measured in the blood of patients with obesity requiring bariatric surgery to control their weight, health, and quality of life. The markers of oxidative stress we measured were GSH (reduced glutathione), GSSG (oxidized glutathione), MDA (malondialdehyde), nitrite, and nitrate anions (and their ratio, also). These markers cover a large part of the process called oxidative stress, which describes the imbalance between the production and neutralization of molecules and atoms that contain a single electron in the valence orbitals (definition of free radicals). These reactive species can react with biomolecules, leading to destruction of important cellular structures, or can reduce the availability of antioxidant molecules (also known as spin traps) that are used to protect cells against free radicals that are produced even under the best biochemical and health circumstances [17].

This study confirms previous findings that obesity is associated with increased oxidative stress, and that bariatric surgery can ameliorate several redox-related parameters. Specifically, we found that GSH levels increased, MDA levels decreased, and nitrite/nitrate concentrations were altered one year after surgery.

The explanation for why the following markers were chosen in order to obtain good insight into the oxidative damage suffered by our patients are presented hereunder. It can be seen that a good coverage of the oxidative damage to tissues and the ability of the patient to reduce such damage is achieved by the measurements we made.

### 4.1. Glutathione (GSH/GSSG)

Our observation of increases in reduced glutathione (GSH) postoperatively, alongside an improved GSH/GSSG ratio, suggests enhanced systemic antioxidant capacity following weight loss. This aligns with findings from Choromańska et al. (2020) and Abulmeaty et al. (2023), who also observed redox restoration after bariatric interventions. The observed inverse correlations between ΔBMI and ΔGSH further support the relationship between fat-mass reduction and antioxidant recovery [16,17].

Glutathione blood concentration shows the ability of the human organism to neutralize free radicals (by electron transfer process, but also by directly binding to the free radical), through processes by which glutathione, by enzymatic pathway, can be restored to its original form or the formation of covalent bonds when glutathione is lost and cannot be recovered. A reasonable explanation for this fact could be the increased consumption of glutathione used during massive body-weight loss, giving rise to increased lipid peroxidation processes. Reduced, oxidized, and total glutathione levels serve as markers of oxidative stress [18].

### 4.2. Malondialdehyde

MDA is produced when unsaturated fatty acids, present in the cell membrane, are damaged by the hydroxy free radical process that leads to cell necrosis—a pluricellular event (not only is the affected cell subjected to destruction, but the surrounding cells could also be affected). MDA is an extremely important marker because it induces peroxidation of cell-membrane lipids, associated with cell necrosis. No antioxidant is able to directly reduce the activity of hydroxy free radical, but substances able to reduce its precursors are known. Therefore, reduction in the levels of MDA serves as an evaluation of the antioxidant ability of certain molecules [19].

As a well-established lipid peroxidation marker, MDA’s significant decrease post-surgery supports reduced free radical burden. The improvement in MDA aligns with better mitochondrial efficiency and decreased inflammatory response, typical after sustained weight loss [7].

### 4.3. Nitrite and Nitrate (NO_2_^−^/NO_3_^−^)

Nitrite anion is a marker of nitric oxide formation, a molecule which can have both antioxidant and pro-oxidant activity in the human body. The nitrite/nitrate ratio was recently included in the markers of oxidative stress.

Nitrite is the first decomposition product of nitric oxide (NO). NO serves as an antioxidant in normal circumstances (preventing the cholesterol oxidation process = formation of the pro-atherosclerotic moiety, 25-OH cholesterol, by preventing the superoxide anion reaction with cholesterol). It also serves as coronary dilatator, neurotransmitter, and supports other biological functions. But as a free radical, when archives are higher than physiological (picomolar; pM) concentrations it transforms into nitrogen dioxide (NO_2_), a substance able to nitrate tyrosine residues in proteins, a process known to negatively affect the cardiac muscle. The nitrite/nitrate ratio is also accepted as an indicator of oxidative stress. However, due to the large amounts of nitrate in some types of vegetable foods, such a ratio should be treated with care and never used alone in estimation of oxidative stress [8].

Despite reductions in nitrite and nitrate levels postoperatively, their interpretation is complex. Nitrite serves as a proxy for NO bioavailability, and reductions may reflect diminished systemic NO production or altered endothelial function. However, this change does not necessarily indicate a harmful shift. Similar trends were reported by Wei et al., who emphasized the contextual nature of NO metabolism. Our findings suggest that although oxidative damage is reduced, the balance of NO-related species warrants further exploration. Future work should include endothelial function markers (e.g., flow-mediated dilation or cGMP) to better understand this dynamic [8,15].

As expected, the weight loss achived by the patients improved reduced plasma glutathione concentrations (GSSH), probably due to the reduction in body weight, and the consecutive reduction in the excessive burn of calories due to the presence of excessive body mass. In such situations (excessive body mass = obesity), the metabolic processes will be accelerated when compared with that of an individual with so-called “normal body mass”. Overproduction of free radicals during increased physical exercise is usually counteracted by the so-called “antioxidant” mechanisms. However, studies have shown that low- and moderate-intensity exercises are associated with positive effects on the heart (other effects are not considered here) and excessive training could have similar effects on the heart infarction risk as sedentarism has [20].

The decreased caloric intake that follows bariatric surgery leads to a reduction in the caloric usage of the organism and therefore, a reduction in formation of free radicals. Of course, the reduction in the caloric intake outweighs the reduction in the caloric consumption, therefore an advantage for the reduction in body mass can be seen for a long period of time [21].

Before the surgery, GSH concentrations, as expected, inversely correlated with the BMI of the patient, showing that BMI has an impact either on the production or the consumption of the GSH (lower production associated or not with higher consumption). This is due to the fact that, in such patients with obesity, even a slight effort will require intensification of the metabolic processes at levels that are far beyond those that are acceptable as a physiological response to an increased physical activity. Post-surgery, the changes in the GSH values (ΔGSH) inversely correlated with the changes in the BMI (ΔBMI), showing that improvement in this oxidative stress marker is correlated with therapeutical success (reduction in the BMI—ΔBMI) [22].

The biochemical improvements observed in oxidative stress markers postoperatively likely reflect complex molecular and metabolic adaptations following significant weight loss. The increase in reduced glutathione (GSH) may be attributed to enhanced activity of the Nrf2–ARE (antioxidant response element) signaling pathway, which regulates the transcription of genes responsible for antioxidant defenses such as GCLC and GCLM—enzymes essential for GSH synthesis [23,24]. Weight loss also diminishes systemic inflammation and pro-oxidant cytokines (e.g., TNF-α, IL-6), which are known to suppress Nrf2 activity [25]. In parallel, the reduction in malondialdehyde (MDA), a marker of lipid peroxidation, may result from decreased mitochondrial-ROS production due to improved insulin sensitivity, reduced adipocyte dysfunction, and lower oxidative-metabolism stress [26]. Furthermore, the observed decline in nitrite and nitrate levels suggests a downregulation of inducible nitric oxide synthase (iNOS) activity, a common consequence of resolving chronic low-grade inflammation. This reduction in NO metabolites might also reflect improved endothelial function, favoring eNOS-mediated NO production at physiologic levels rather than pathological overproduction. Together, these findings suggest that bariatric surgery not only reduces oxidative stress by limiting ROS production but also restores regulatory pathways that govern redox homeostasis [27].

Our observation that bariatric surgery leads to reductions in BMI, venous plasma glucose, and oxidative stress markers aligns with existing literature not only in magnitude but in mechanistic interpretation. In particular, Jakubiak et al. used principal-component analysis in a large cohort to show that among the components of metabolic syndrome, the cluster defined by ‘obesity and insulin resistance’ exhibited the strongest association with oxidative stress parameters. Thus, our results support the hypothesis that the interplay of adiposity and insulin resistance is a principal driver of oxidative imbalance—and that surgical weight-loss mitigates oxidative stress largely through this axis [28].

While our biomarker results are not novel per se, this study provides valuable regional insights by presenting data from a Romanian cohort—a population underrepresented in bariatric redox studies. Thus, the findings may inform region-specific health strategies or dietary interventions. To our knowledge, few studies have simultaneously assessed this panel of oxidative stress markers in this demographic context [11,17].

### 4.4. Strengths and Limitations

This study has several strengths. First, it is based on a relatively large and well-defined cohort, including both patients with obesity undergoing bariatric surgery and a matched control group of normoponderal surgical patients. The prospective design, with a one-year follow-up, allowed us to evaluate longitudinal changes in oxidative stress biomarkers within the same individuals, strengthening causal inference. Furthermore, we applied validated HPLC-based analytical methods for quantification of glutathione, malondialdehyde, nitrite, and nitrate, providing sensitive and specific biochemical measurements.

However, some limitations should also be acknowledged. The study was conducted at two centers in a single region of Romania, which may limit generalizability to other populations. Although we included 50 patients per group, larger multicenter cohorts would provide greater statistical power, especially for subgroup analyses. We did not perform additional mechanistic assessments (e.g., measurement of antioxidant enzyme activity, endothelial function, or inflammatory signaling pathways), which could have provided deeper insights into the observed biochemical changes. Moreover, while follow-up was limited to one year, longer-term studies are needed to determine the durability of redox-homeostasis improvements after bariatric surgery. Finally, dietary factors, physical activity, and medication use were not systematically controlled postoperatively, and these may have influenced oxidative-stress status.

Finally, translational studies exploring how improvements in oxidative stress relate to long-term clinical outcomes—such as reduced cardiovascular risk, better metabolic control, and improved quality of life—are needed to clarify the broader health benefits of bariatric surgery.

## 5. Conclusions

Our study, conducted with a significant number of patients, shows that weight loss could bring significant improvements in certain biomarkers of oxidative stress (glutathione), but could also negatively affect other parameters such as nitrite (a good biomarker of NO formation). Further studies are required to establish if weight loss after bariatric surgery has desirable or undesirable effects on the patients and what measures could be undertaken to ensure only positive effects are experienced.

## Figures and Tables

**Table 1:** Time-table of the gradient elution in the case of GSH determination [12].

Time (min)	Phosphate Buffer 20 mM, pH = 2.5 (%)	Acetonitrile (%)	Flow (mL/min)
0	98	2	1
19	50	50	1
19.1	98	2	1
21	98	2	1

**Table 2:** Time-table of the gradient elution for nitrate and nitrite determination [14].

Time (min)	Tetrabutylammonium Hydroxide (5 mM, pH 2.5) (%)	Acetonitrile (%)	Methanol (%)	Flow (mL/min)
0	92	8	0	1
8.5	92	8	0	1
10.5	48	8	44	1
15.5	48	8	44	1
15.6	92	8	0	1
18.5	92	8	0	1
18.6	92	8	0	1
22.0	92	8	0	1
23.0	92	8	0	1

**Table 3:** Baseline data of all study groups.

Parameter	Bariatric Group (B1/Preoperative)	Bariatric Group (B2/Postoperative)	Control Group (CG)	*p*-Value
GENDER (FEMALE%)	40 (80.00%)	40 (80.00%)	33 (66.0%)	0.082
AGE (years)	44.28 ± 12.53	45.60 ± 11.61	47.82 ± 15.05	0.398
BMI	45.70 ± 6.85	31.75 ± 4.70	24.04 ± 1.70	<0.001
CA (cm)	131.12 ± 20.09	101.32 ± 13.28	84.80 ± 8.57	<0.001
HEIGHT (cm)	166.02 ± 7.13	166.18 ± 6.19	168.44 ± 8.15	0.175
WEIGHT (kg)	126.26 ± 22.11	87.84 ± 14.84	68.44 ± 8.91	<0.001
GGT (U/I)	30.10 ± 12.01	28.62 ± 25.50	32.86 ± 25.03	0.615
GLUCOZA (mg/dL)	112.89 ± 30.04	99.49 ± 18.27	99.12 ± 15.45	0.002
GOT (UI/I)	28.90 ± 28.78	23.81 ± 17.52	24.44 ± 14.93	0.429
GPT (UI/I)	37.70 ± 36.31	24.42 ± 16.72	24.41 ± 11.04	0.007
HGB (g/dL)	13.90 ± 1.42	13.51 ± 1.57	13.60 ± 1.62	0.423
HTC (%)	41.87 ± 3.62	40.31 ± 4.26	39.87 ± 5.18	0.062
LEUKOCYTES	8548.80 ± 2079.61	7693.36 ± 2236.39	7074.22 ± 1813.16	0.002
NEUTROPHILS	5341.40 ± 1620.59	4853.00 ± 2054.18	4461.20 ± 1473.78	0.042
LYMPHOCYTES	2538.80 ± 685.63	2369.80 ± 605.79	2019.00 ± 682.27	<0.001
EOSINOPHILS	207.20 ± 153.02	165.92 ± 102.75	162.00 ± 141.64	0.179
MONOCYTES	583.80 ± 350.54	746.28 ± 987.20	510.80 ± 182.90	0.149
TROMBOCYTES	299.78 ± 79.90	260.74 ± 72.61	241.58 ± 68.81	<0.001
HDL (mg/dL)	51.86 ± 16.93	59.67 ± 23.57	55.63 ± 6.72	0.079
TOTAL CHOLESTEROL (mg/dL)	185.62 ± 44.15	187.94 ± 51.54	177.46 ± 41.87	0.491
LDL (mg/dL)	121.64 ± 33.79	127.10 ± 42.16	128.93 ± 36.68	0.604
TG (mg/dL)	149.92 ± 59.94	120.57 ± 44.30	101.08 ± 31.17	<0.001
GSH/GSSG	0.77 ± 0.19	0.79 ± 0.17	0.92 ± 0.18	<0.001
MDA (ng/mL)	16.62 ± 8.34	21.58 ± 13.91	14.77 ± 12.17	0.014
NO_2_ (ug/mL)	22.18 ± 16.63	17.15 ± 12.20	12.68 ± 16.78	0.041
NO_3_ (ug/mL)	11.70 ± 6.54	18.61 ± 2.35	19.97 ± 2.47	<0.001
ug GSH total/mL	8.44 ± 2.14	8.38 ± 1.80	10.19 ± 1.89	<0.001
ug GSH/mL	3.51 ± 0.67	3.59 ± 0.51	4.78 ± 0.67	<0.001
ug GSSG/mL	4.92 ± 1.71	4.79 ± 1.46	5.42 ± 1.44	0.112

## Data Availability

The datasets used and analyzed during the current study are available from the corresponding author on reasonable request.

## Abbreviations

The following abbreviations are used in this manuscript:

25-OH	25-hydroxy
BDW	Body dry weight
BH	Body height
BMI	Body mass index
BW	Body weight
EDTA	Etilendiaminotetraacetic acid
Fe^2+^	Iron two
G_tot_	Total glutathione
GGT	Gamma-glutamyl transferase
GOT	Aspartate aminotransferase
GPT	Alanine aminotransferase
GSH	Reduced glutathione
GSSG	Oxidized glutathione
H_2_O_2_	Hydrogen peroxide
H_2_SO_4_	Sulfuric acid
HDL	High density lipoprotein
HGB	Hemoglobin
•OH	Hydroxide ion
HPLC	High-performance liquid chromatography
HTC	Hematocrit
HTN	Hypertension
LDL	Low-density lipoprotein
LSG	Sleeve gastrectomy
MDA	Malondialdehyde
MAFLD	Metabolic-associated fatty liver disease
NO	Nitric oxide
NO_2_	Nitrogen dioxide
NO_2_^−^	Nitrite
NO_3_^−^	Nitrate
OAGB	One-anastomosis gastric bypass
OS	Oxidative stress
OSA	Obstructive sleep apnea
ROS	Reactive oxygen species
RP	Reverse phase
RNS	Reactive nitrogen species
SOD	Superoxide dismutases
T2DM	Type 2 diabetes
TG	Triglyceride
WC	Waist circumference
